# A Case of Ischemic Stroke With Congenital Protein C Deficiency and Carotid Web Successfully Treated by Anticoagulant and Carotid Stenting

**DOI:** 10.3389/fneur.2020.00099

**Published:** 2020-02-18

**Authors:** Sadayoshi Watanabe, Shoji Matsumoto, Ichiro Nakahara, Akira Ishii, Taketo Hatano, Minako Mori, Eriko Morishita, Izumi Nagata

**Affiliations:** ^1^Department of Comprehensive Strokology, Fujita Health University School of Medicine, Aichi, Japan; ^2^Department of Neurosurgery, Kyoto University Graduate School of Medicine, Kyoto, Japan; ^3^Department of Neurosurgery, Stroke Center, Kokura Memorial Hospital, Fukuoka, Japan; ^4^Department of Hematology and Oncology, Kyoto University Graduate School of Medicine, Kyoto, Japan; ^5^Department of Clinical Laboratory Sciences, Graduate School of Medical Science, Kanazawa University, Kanazawa, Japan

**Keywords:** protein C deficiency, thrombus formation, carotid web, cerebral infarction, carotid artery stenting

## Abstract

**Objective:** A rare case of thromboembolic cerebral infarction due to carotid web in a patient with congenital protein C deficiency is reported.

**Case Presentation:** A patient in her 40's with left-side hemiparesis was transferred to our hospital under continuous intravenous injection of heparin. Magnetic resonance angiography demonstrated occlusion of the right middle cerebral artery (MCA). Conventional angiography revealed recanalization of the right MCA and a carotid web at the origin of the right internal carotid artery. Ultrasound scan of the carotid artery on the 19th day revealed thrombus formation on the distal portion of the carotid web. We performed carotid artery stenting to prevent thrombus formation by suppressing the carotid web to the vessel wall and by regulating the turbulent flow. The patient had no recurrence of stroke under-anticoagulation with warfarin during the 2-year follow-up period.

**Conclusion:** To our knowledge, this is the first report in which an immediate thrombus formation on the carotid web was observed in a patient with congenital protein C deficiency. In a case of acute ischemic stroke with carotid web, especially when congenital coagulopathy such as protein C deficiency is suspected, careful follow-up with ultrasound imaging should be performed.

## Introduction

Carotid web is a shelf-like projection in the lumen of the internal carotid artery bulb and is recognized as a cause of stroke. However, the optimal treatment for symptomatic carotid web has not been thoroughly investigated (1). Moreover, congenital protein C deficiency induces thrombogenicity and also increases the risk of arterial thromboembolism, especially in young patients. There is no report on the clinical course and treatment strategy for a case complicated by both carotid web and protein C deficiency. Here, we report a case of thromboembolic cerebral infarction in which protein C deficiency was thought to accelerate thrombus formation on the carotid web. We confirmed thrombus formation with carotid ultrasound.

## Case Presentation

A patient in her 40's with a wake-up stroke resulting in left-side hemiparesis was admitted to a previous hospital. The patient's head CT showed no evidence of cerebral hemorrhage, and ischemic stroke was suspected. The patient was then transferred to our hospital under continuous intravenous injection of low-dose unfractionated heparin (5,000 U intravenously, followed by continuous infusion of 500 U/h) prescribed by the physician of the previous hospital. Her past medical history was unremarkable, and she was under no medications. She did not use tobacco or thrombogenic drugs such as steroids or oral contraceptives. She had a negative family history for stroke. Her pulse was 90 beats per minute, pulse rhythm was regular, and blood pressure was 160/80 mmHg. She did not have any history of atrial fibrillation (AF). Her general physical examination results were normal. Neurological examinations revealed eye deviation to the right, severe inattentiveness on the left side, and left-side hemiparesis with Manual Muscle Testing scores of 1/5 for her left arm and 4/5 for her left leg. Laboratory studies yielded the following results: white blood cell count, 13,200/μl; C-reactive protein, 0.2 mg/dl; LDL-cholesterol, 83 mg/dl; fibrin/fibrinogen degradation products, 0.0 μg/ml; D-dimer, 0.2 μg/ml; prothrombin time-international normalized ratio, 1.32; and activated partial thromboplastin time, 100 s (normal range: 25.0–38.0 s). Protein C activity was 45% (normal range: 64–146%), total protein S antigen was 78% (normal range: 60–150%), and antithrombin activity was 84% (normal range: 80–120%). Tests for lupus anticoagulant, anti-cardiolipin antibodies, and anti-cardiolipin-beta 2-glycoprotein I complex antibody were negative.

**Figure 1:**
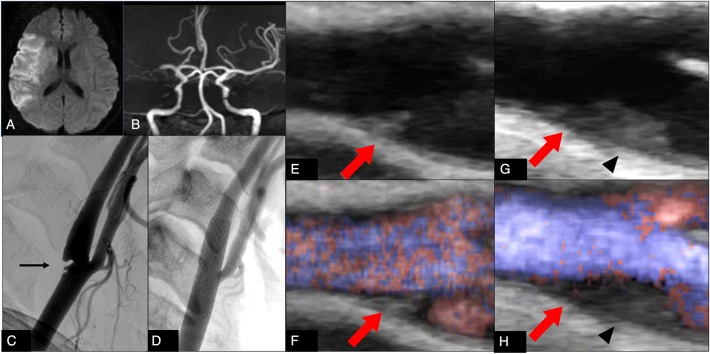
**(A)** Diffusion-weighted magnetic resonance imaging on admission shows a hyperintense area in the territory of the right middle cerebral artery. **(B)** Magnetic resonance angiography on admission shows right middle cerebral artery occlusion. **(C)** Digital subtraction angiography on admission shows a carotid web (arrow) at the origin of the right internal carotid artery. **(D)** Digital subtraction angiography after carotid artery stenting on the 32nd day shows disappearance of the web protruding into the carotid artery lumen. **(E,F)** Ultrasonography on the 2nd day shows a carotid web (arrow) at the origin of the right internal carotid artery (**E**: brightness mode, **F**: B-flow mode). **(G,H)** Ultrasonography on the 19th day shows a carotid web (arrow) and new thrombus formation on the distal portion of the carotid web (arrowhead) (**G**: brightness mode, **H**: B-flow mode).

Head magnetic resonance imaging (MRI) demonstrated acute cerebral infarction in the right middle cerebral artery (MCA) territory (Figure 1A) and magnetic resonance angiography (MRA) demonstrated occlusion of the main trunk of the right MCA (Figure 1B).

Because 7 h had passed since her condition had been confirmed normal, we attempted endovascular therapy. However, conventional angiography revealed recanalization of the right MCA and a carotid web at the origin of the right internal carotid artery, which showed approximately 30% stenosis according to the North American Symptomatic Carotid Endarterectomy Trial criteria (Figure 1C).

## Clinical Course After Admission

On the second day, carotid duplex Doppler ultrasound confirmed the angiographic diagnosis (Figures 1E,F). Ultrasound examination was performed on a Logiq 7 pro (GE healthcare, Illinois, Chicago, USA) with a linear array transducer (10 L probe).

We initiated antiplatelet therapy (clopidogrel 75 mg/day and aspirin 100 mg/day) and anticoagulant therapy for a short duration (argatroban, 60 mg/day for 2 days followed by 20 mg/day for 5 days). Subsequently, we stopped anticoagulant therapy and continued with only antiplatelet therapy. However, the follow-up ultrasound scan of the carotid artery on the 19th day revealed thrombus formation on the distal portion of the carotid web (Figures 1G,H). We restarted anticoagulant therapy (continuous infusion of 500 U/h heparin, followed by 4 mg/day warfarin). On the 29th day, follow-up carotid ultrasound scanning showed a decrease of the thrombus on the distal portion of the carotid web. There was no new ischemic lesion on MRI. After obtaining informed consent from the proband, genetic analysis was done. The genetic analysis identified a single-base substitution, c.631G>A; p.Arg211Trp (protein C Tochigi), demonstrating a heterozygosity of the proband for congenital protein C deficiency (2). Neither persistent nor paroxysmal AF was detected by 24-h Holter monitoring. Transesophageal echocardiography showed no embolic source in her heart or aorta. Ultrasonography of the lower extremities showed no deep vein thrombosis. Therefore, we speculated protein C deficiency to have promoted thrombus formation on the web when anticoagulation therapy was stopped, and we initially proposed carotid endarterectomy to eliminate the web, providing a scaffold for thrombus formation. However, because the patient refused surgery, we changed our plan to perform carotid artery stenting (CAS) to prevent thrombus formation by regulating turbulent flow by suppressing the protruding web into the carotid wall.

She underwent CAS on the 32nd day using a closed stent under proximal and distal protection, with successful results and no complications (Figure 1D). She was discharged home with dexterity movement disorder in the right hand on the 50th day and had no recurrence of stroke while under anticoagulant therapy with warfarin during the 2-year follow-up period.

We have obtained a written informed consent from the patient. This study was approved by the Kokura Memorial Hospital Human Genome and Gene Analysis Research Ethics Committee.

## Discussion

We report a case of thromboembolic cerebral infarction in which protein C deficiency was thought to accelerate thrombus formation on the distal portion of the carotid web. No recurrence of stroke was noted for 2 years after carotid stenting with anticoagulant therapy with warfarin.

Carotid web is a shelf-like intraluminal projection within the internal carotid artery bulb and is referred to as a type of fibromuscular dysplasia (3). It has been reported that the prevalence of carotid web ranges from 9.4 to 37% in cases of cryptogenic stroke of anterior circulation (4, 5). Moreover, carotid web is recognized as a cause of recurring ischemic stroke (6), the etiology of which is thought to be the web projecting into the carotid arterial lumen, thus providing a scaffold for thrombus formation and creating turbulent flow or flow stasis promoting blood coagulation and resulting in thrombus formation (7). This thrombus on the web, when scattered distally by the blood flow, causes thromboembolic stroke. Our case showed an immediate increase in thrombus formation on the web when anticoagulant therapy was interrupted.

Protein C serves as the main anticoagulant factor responsible for the negative feedback in the blood coagulation cascade. Protein C-Tochigi houses one of the main gene mutations (p.Arg211Trp) in the Japanese population and does not function as an anticoagulant factor, thus resulting in protein C deficiency (2). Protein C deficiency induces a hypercoagulable state and mainly causes venous thromboembolism(VTE), including deep vein thrombosis, pulmonary thromboembolism, and mesenteric vein thrombosis (8). It has been reported that, in protein C deficiency, the incidence of VTE is frequent but that of arterial thrombosis is relatively rare (8–10). However, protein C deficiency contributes to the younger onset of arterial thromboembolism (11, 12). Moreover, in recent years, it has been reported that protein C deficiency has a strong association with arterial thromboembolism, especially in the presence of additional vascular risks such as diabetes mellitus (13).

Our case was thought to be associated with an extremely high risk of recurrent stroke because of both carotid web, which is a high-risk lesion related to recurrent stroke, and protein C deficiency, which causes a hypercoagulable state. Here, we confirmed rapidly increasing thrombus formation at the distal portion of the carotid web using carotid ultrasound despite the provision of antiplatelet therapy after discontinuation of anticoagulant therapy. If the additional ultrasonic examination had not been conducted, we would not have restarted anticoagulant or considered surgical therapy, because we would not have noticed the increase in thrombus formation. As a result, there would have been a high possibility of recurrence of cerebral infarction.

The optimal management strategy to prevent ischemic stroke in patients with both carotid web and protein C deficiency remains unknown. As for carotid web, Joux et al. reported that the recurrence rate of ischemic stroke was 0% in patients in whom it had been surgically removed and 30% in patients receiving alternative medical therapy (14). Haussen et al. performed sixteen CAS for symptomatic carotid web and observed no recurrent stroke for the median follow up of 4 months. (6) As in our case, recurrence of stroke has not been observed for 2 years after CAS under anticoagulant therapy with warfarin, and carotid stenting and anticoagulant therapy may be an effective treatment option to prevent ischemic stroke in patients with both carotid web and protein C deficiency. However, careful follow-up is needed because protein C deficiency can cause in-stent thrombus formation after carotid stenting (15). To our knowledge, this is the first report in which thrombus formation on the carotid web following cessation of anticoagulant therapy was confirmed by carotid ultrasonography in a patient with protein C deficiency. In the case of acute ischemic stroke with carotid web, especially when congenital coagulopathy such as protein C deficiency is suspected, careful follow-up with ultrasound imaging should be conducted. A limitation of this report is that only one case was reported, and the follow-up period was relatively short. Long-term follow-up should be performed to confirm the efficacy of our treatment strategy for prevention of lifetime recurrent stroke. Further studies should be accumulated to confirm the optimal treatment for cases with carotid web and congenital coagulopathy.

## Ethics Statement

Written informed consent was obtained from the individual(s) for the publication of any potentially identifiable images or data included in this article.

## Author Contributions

SW, SM, and INak contributed to the conception and design of this paper. SW, SM, INak, AI, TH, and INag contributed to drafting the text and preparing the figures. MM and EM contributed to sequence analysis of the deoxyribonucleic acid sample of this case.

### Conflict of Interest

SM reports personal fees from Kaneka Medica, Terumo, Nipro and grants from Grant-in-Aid for Scientific Research (Grant No. 16K10727), outside the submitted work. INak reports personal fees from Kaneka Medica, Terumo, Nipro, outside the submitted work. AI reports personal fees from Medtronic Japan, Stryker, outside the submitted work. TH reports personal fees from Kaneka Medica, Pfizer, Bayer, Medtronic Japan, Bristol-Myers Squibb, Daiichi Sankyo, Sanofi, Otsuka, Selenovus, Stryker, outside the submitted work. EM reports grants from Ministry of Health, Labor and Welfare of Japan (Grant No. 6046619-01), Ministry of Education, Culture, Sports, Science and Technology of Japan (Grant No. 18K07442), Japan Agency for Medical Research and Development (Grant No. 17ek0109210h0001), outside the submitted work. INag reports personal fees from Bristol-Myers Squibb, outside the submitted work. The remaining authors declare that the research was conducted in the absence of any commercial or financial relationships that could be construed as a potential conflict of interest.

## References

[1] Kim SJ Nogueira RG Haussen DC. Current understanding and gaps in research of carotid webs in ischemic strokes: a review. JAMA Neurol. (2018) 76:355–61. 10.1001/jamaneurol.2018.3366 30398546

[2] Matsuda M Sugo T Sakata Y Murayama H Mimuro J Tanabe S. A thrombotic state due to an abnormal protein C. N Engl J Med. (1988) 319:1265–8. 10.1056/NEJM198811103191907 3185623

[3] Sajedi PI Gonzalez JN Cronin CA Kouo T Steven A Zhuo J. Carotid bulb webs as a cause of “cryptogenic” ischemic stroke. Am J Neuroradiol. (2017) 38:1399–404. 10.3174/ajnr.A5208 28495950 PMC7959897

[4] Coutinho JM Derkatch S Potvin AR Tomlinson G Casaubon LK Silver FL. Carotid artery web and ischemic stroke: a case-control study. Neurology. (2017) 88:65–69. 10.1212/WNL.0000000000003464 27864523 PMC5200857

[5] Joux J Boulanger M Jeannin S. Association between carotid bulb diaphragm and ischemic stroke in young afro-caribbean patients: a population-based case-control study. Stroke. (2016) 47:2641–4. 10.1161/STROKEAHA.116.013918 27625379

[6] Haussen DC Grossberg JA Bouslama M Pradilla G Belagaje S Bianchi N. Carotid web (Intimal Fibromuscular Dysplasia) has high stroke recurrence risk and is amenable to stenting. Stroke. (2017) 48:3134–7. 10.1161/STROKEAHA.117.019020 29018133

[7] Choi PM Singh D Trivedi A Qazi E George D Wong J. Carotid webs and recurrent ischemic strokes in the Era of CT angiography. AJNR Am J Neuroradiol. (2015) 36:2134–9. 10.3174/ajnr.A4431 26228877 PMC7964886

[8] Allaart CF Poort SR Rosendaal FR Reitsma PH Bertina RM Briët E. Increased risk of venous thrombosis in carriers of hereditary protein C deficiency defect. Lancet. (1993) 341:134–8. 10.1016/0140-6736(93)90003-Y 8093743

[9] Pabinger I Kyrle PA Heistinger M Eichinger S Wittmann E Lechner K. The risk of thromboembolism in asymptomatic patients with protein C and protein S deficiency: a prospective cohort study. Thromb Haemost. (1994) 71:441–5. 10.1055/s-0038-1642457 8052960

[10] De Stefano V Leone G Micalizzi P Teofili L Falappa PG Pollari G Arterial thrombosis as clinical manifestation of congenital protein C deficiency. Ann Hematol. (1991) 62:180–3. 10.1007/BF01703145 2049465

[11] Sakata T Kario K Katayama Y Matsuyama T Kato H Miyata T. Analysis of 45 episodes of arterial occlusive disease in Japanese patients with congenital protein C deficiency. Thromb Res. (1999) 94:69–78. 10.1016/S0049-3848(98)00194-7 10230891

[12] Mahmoodi BK Brouwer JL Veeger NJ van der Meer J. Hereditary deficiency of protein C or protein S confers increased risk of arterial thromboembolic events at a young age: results from a large family cohort study. Circulation. (2008) 118:1659–67. 10.1161/CIRCULATIONAHA.108.780759 18824642

[13] Mahmoodi BK Veeger NJ Middeldorp S Lijfering WM Brouwer J-LP Berg JT. Interaction of hereditary thrombophilia and traditional cardiovascular risk factors on the risk of arterial thromboembolism: pooled analysis of four family cohort studies. Circ Cardiovasc Genet. (2016) 9:79–85. 10.1161/CIRCGENETICS.115.001211 26679867

[14] Joux J Chausson N Jeannin S Saint-Vil M Mejdoubi M Hennequin JL. Carotid-bulb atypical fibromuscular dysplasia in young Afro-Caribbean patients with stroke. Stroke. (2014) 45:3711–3. 10.1161/STROKEAHA.114.007313 25358695

[15] Iwamoto Y Kitano T Matsubara S Uno M Yagita Y. In-stent thrombosis after carotid artery stenting in a patient with protein C deficiency. Neurol Sci. (2018) 39:2229–30. 10.1007/s10072-018-3544-6 30145678

